# Clinical outcomes of pretarsal fullness formation by capsulopalpebral fascia plication with silicone prosthesis implantation and pretarsal fullness surgery: Retraction

**DOI:** 10.1097/MD.0000000000050456

**Published:** 2026-09-04

**Authors:** Xiaobo Zhu, Shanzhi Zhu, Jun Yao, Kejia Wang

The article, “Clinical outcomes of pretarsal fullness formation by capsulopalpebral fascia plication with silicone prosthesis implantation and pretarsal fullness surgery,”^[1]^ which appears in Volume 105, Issue 28 of *Medicine*, is being retracted at the request of the authors after an internal post-publication review identified critical inaccuracies in both the clinical baseline timeline and partial technical descriptions, which arose from an inadvertent administrative oversight during the final manuscript submission and revision process and resulted in misrepresented case enrollment timing and inconsistent surgical technical descriptions in the clinical series. All coauthors agreed to this retraction.
